# Exploring the association between agricultural production systems and household diets in Viet Nam

**DOI:** 10.1007/s12571-022-01276-x

**Published:** 2022-04-27

**Authors:** Arkadeep Bandyopadhyay, Carlo Azzarri, Beliyou Haile, Chungmann Kim, Cristina Alvarez, Ana Moltedo, Abdul Sattar, Winnie Bell, Beatrice L. Rogers

**Affiliations:** 1 International Food Policy Research Institute (Environment and Production Technology Division), Washington, DC USA; 2 Department of Agricultural Economics, University of Illinois at Urbana-Champaign, Champaign, IL USA; 3 Food and Agriculture Organization, Rome, Italy; 4 Friedman School of Nutrition Science and Policy, Tufts University, Medford, MA USA

**Keywords:** Production systems, Balanced diet, Viet Nam, Macronutrients

## Abstract

The government of Viet Nam promotes an integrated and diversified production system that focuses on the symbiotic relationship of livestock, aquaculture, and fruits and vegetables (F&V), locally known as *Vuon Ao Chuong* (VAC). The expectation is that this system can prevent soil degradation, while improving dietary quality and income. This study examines the correlation between VAC production systems and diets using cross-sectional data from the 2016 round of the Viet Nam Household Living Standards Survey (VHLSS). Using ordinary least squares, we model four continuous outcome variables related to quantity consumed of fruits and vegetables, fiber, animal protein, and dietary energy; while using logistical regression, we model three indicator variables related to whether diets are balanced in terms of intake of dietary energy derived from carbohydrates, proteins, and fats. While individual components of VAC, such as aquaculture or F&V production, show a positive correlation with one or more dietary indicators, adoption of the full VAC system is found to be positively correlated only with dietary fiber consumption, making it challenging to establish a causal link between system adoption and improved dietary quality. However, we find that several socioeconomic variables, such as access to markets, household wealth, education of the household members, and household size are positively associated with one or more dietary indicators. Further research is needed to establish strong and causal relationships, or lack thereof, between VAC system and diets by exploiting the panel structure of VHLSS to examine the role of VAC in improving nutritional outcomes in Viet Nam.

## Introduction

The agricultural sector in Viet Nam has made considerable progress over the past 25 years. Consistent development in smallholder rice productivity and intensification through the 1990s have played a pivotal role in reducing poverty, improving food security and strengthening social stability (World Bank, 2016). However, this enhanced output (crops and livestock) has come at an ever-increasing environmental cost (Anh et al., 2010; Hoai et al., 2011; Nguyen, 2017), a consequence of increased usage of natural resources combined with a heavy reliance on fertilizer and other associated agro-chemicals (World Bank, 2016). Mindful of these limitations, Viet Nam’s future strategy in boosting agricultural production would require greater emphasis on producing more with less – employing fewer natural resources and damaging inputs.

Viet Nam’s current status as a food secure nation (FAO et al., 2020)^1^ has been preceded with episodes of acute food shortage and scarcity (Butterfield, 1977; Chapman, 1979; Crossette, 1988; Huff, 2019) during which, unsurprisingly, the rural poor were affected the most. In the face of prolonged food insecurity which lasted even after the Viet Nam war, the government enacted the *Doi Moi* (renovation) policy in 1986, which abandoned the Soviet model of collectivization of farms and increased the prevalence of market-oriented production (Beresford, 2008). This ushered in an era of vigorous economic growth accompanied with poverty reduction. Indeed, the poverty headcount ratio at $1.9 per day at 2011 Purchasing Power Parity (PPP)^2^ has decreased from 52.9% in 1992 to 2% in 2016 (World Bank, 2020).

Together with economic prosperity, dietary habits of the nation have changed accordingly. While the share of calories derived from protein has remained relatively stable between 2004 and 2014, those derived from fats and carbohydrates have increased by more than 33% and decreased by more than 7% respectively (Trinh & Morais, 2017). Owing to this increased reliance on fat and energy dense food, Viet Nam has experienced an uptick in obesity and other non-communicable diseases (NCDs) (Trinh Thi et al., 2018). Along with 6.4% of the total population being undernourished, and with stunting and wasting rates among children below 5 years of age at 23.8% and 5.8% respectively (FAO et al., 2020), Viet Nam is facing the issue of the double burden of over- and under- nutrition, similar to the trends observed in many poor and emerging economies (Abdullah, 2015).

Key agricultural commodities include rice (grown mainly in the Red and Mekong river deltas), maize, manioc, sweet potato, nuts (grown across the country), tea, coffee (grown in the Central highlands), rubber (grown in the central highlands and southern terrace regions), bananas, and coconuts (grown in the Mekong delta and southern terrace regions) (Buttinger et al., 2021). The nation also exports seafood such as shrimp, squid and crab, which serves as a source of foreign exchange. Freshwater fisheries are located on the plans of the Mekong and Champasak rivers (Buttinger et al., 2021).

Sixty-five percent of the population live in rural areas, with the agricultural sector employing 15% of the entire country’s workforce (CIA, 2016). Due to agricultural intensification and expansion, cropped areas have expanded from 7.3 to 8.9 million hectares (ha) between 1995 and 2014 (GSO, 2016). Cereal (overwhelmingly rice) yield has been steadily increasing for the last several decades, although it has declined marginally in recent years; in 2016 productivity stood at 5,448 kg/ha compared to 5,601 kg/ha in 2015 (World Bank, 2018).

Viet Nam has a strong history of integrated farming. Prior to the *Doi Moi* policy, integrated farming was recognized as an important tool in family food production for the rural poor in the country. The integration of the home lot, garden, livestock and fishpond is referred to as the VAC system (*Vuon* = *garden, Ao* = *pond, Chuong* = *livestock pen*). The garden encompasses the production of various plants bearing fruits and vegetables; the shed includes livestock production; and, finally, the fishpond provides aquaculture production, mainly fish and shrimp. Each element of this production system provides different food items for human consumption. In addition, the three elements are also complementary, where the fishpond acts as a source of water and mud for the garden, thereby nourishing it; the garden provides feed for fish and livestock; and the fishpond serves as a source of animal feed with animal stool complementarily used as fish feed or fertilizer for the garden. Although widespread promotion of the system only began in 1980s, in the 1960s late president Ho Chi Minh had emphasized the importance of small-scale family farm integration (Thanh Luu, 2001). While originally the objective of the movement was to augment and support diets of the rural poor, in light of the growing environmental concerns surrounding agricultural pollution, VAC is also expected to regenerate farm soil quality (Pham Van, 2010). By the early 2000s, 85–90% of rural families maintained a garden and livestock pen, with 30–35% of these households also having fishponds. VAC also played a critical role in supporting and diversifying farm incomes, with 30–60% of income of village households coming from the system (Thanh Luu, 2001).

In this study, we assess and quantify the correlation between the VAC production system, its components, and some selected dietary outcomes, which are complementary in nature and reflective of various aspects of a healthy diet. This entails estimating the association between VAC production system and per capita consumption of fruits and vegetables, fiber, animal protein and dietary energy while controlling for demographic and socioeconomic factors. Recognizing the importance of macronutrient balance (Carreiro et al., 2016; Chow & Hall, 2008; Studnicki et al., 2019), we also look at the correlation between dietary energy consumption from macronutrients and the VAC production system, based on internationally recommended balances for adults suggesting that 55–75%, 15–30% and 10–15% of dietary energy should be derived from carbohydrates, fats, and protein respectively (WHO, 2003).

### Literature

Healthy diets help to protect against malnutrition in all its forms and NCDs that can have a nutritional etiology (e.g. diabetes, heart disease, stroke, and cancer) (World Health Organization, 2003, 2020). Generally, recommendations for a healthy diet include the consumption of a great variety of unprocessed or minimally processed foods; limited consumption of highly processed food and beverages; consumption of whole grains, legumes, nuts, and F&V; and consumption of small amounts of red meat and moderate amounts of other animal-source food (ASF) such as eggs, dairy, poultry, and fish (World Health Organization, 2020). Nevertheless, the exact make-up of a healthy diet varies depending on food items available, dietary customs, and individual characteristics such as age, sex, and degree of physical activity and health priorities of a particular group. For example, Viet Nam’s food-based dietary guidelines emphasize consumption of protein-rich foods from animal and plant sources (FAO, 2013).

In this regard, the importance of F&V consumption cannot be overstated. F&V are a significant source of vitamins, minerals and dietary fiber (Kader, 2001). F&V consumption is strongly linked with improved health outcomes (Ortega, 2006), with antioxidants contained in F&V proven to protect against age-related ailments (Steinberg, 1991). Routine and habitual consumption of F&V is also found to reduce the risk of several common cancers (oral, lung, stomach, and colon) (Steinmetz & Potter, 1991). Despite these benefits, many low- and middle-income countries (LMICs) exhibit concerningly low F&V consumption ─ less than five servings a day or 400 g per day set by the FAO & WHO (FAO & WHO, 2004). During 2002–2003, F&V consumption inadequacy (percentage of population with consumption lower than 400 g/day) across the world ranged from 36.6% (Ghana) to 99.2% (Pakistan) for men; and from 38.0% (Ghana) to 99.3% (Pakistan) for women. In Viet Nam the corresponding figures were 86.7% and 81.8% for men and women, respectively (Hall et al., 2009). Similar to F&V, consumption of dietary fiber may reduce the risk of cardiovascular, infectious and respiratory diseases (Park et al., 2011) and some cancers (Glanz et al., 1994; Goodman et al., 1997). There is also evidence to suggest that prebiotic fibers enhance immune function (Anderson et al., 2009).

Protein is an important macronutrient that provides energy, supports cognitive function, and is necessary for building, maintaining, and repairing tissues, cells, and organs throughout the human body. Protein deficiency has been linked with several health risk factors, especially among pregnant women and children, eventually resulting in diseases such as marasmus and kwashiorkor in extreme cases. Severe protein deficiency is also linked with fatty degeneration of the liver and heart along with the degradation of the small bowel, leading to loss of absorption and digestion capacity (Muller & Krawinkel, 2005). While protein can be obtained from plants, animal sourced protein are more digestible and usable by the human body (Ghosh et al., 2012).

Several socioeconomic and demographic factors are correlated with dietary quality. For example, adult diet quality improves with income (Hiza et al., 2013). Evidence from West Africa suggests that household socioeconomic status is positively associated with increasing intake of dietary energy, fat, sugars and protein, as opposed to other macronutrients (Bosu, 2015). In East Africa, higher agricultural incomes and production diversification are associated to higher quality diets in Malawi (Jones, 2016). Higher education is also correlated with improved food purchasing behavior, given its strong association with income (Turrell & Kavanagh, 2006). Parental, and more specifically maternal, education plays a significant role in the diets of children growing up in the household (Alderman & Headey, 2017; Frost et al., 2005). Higher education of the household head was found to improve household and individual-level dietary diversity in urban Burkina Faso (Becquey et al., 2012), and findings from Bangladesh suggest that intrahousehold gender dynamics and bargaining over resources affects individual dietary quality in the household (Rashid et al., 2011). Women’s bargaining power is also found to be significantly associated with improved child nutrition outcomes (Lépine & Strobl, 2013). In terms of production, crop diversification has been found to have positive impact on crop income and dietary diversity (Pellegrini & Tasciotti, 2014) as well as higher quality diets (Jones, 2016).

Market access can also have a significant effect on the types of foods that are accessible to the household (Koppmair et al., 2017; Larsen & Gilliland, 2009). Where markets are incomplete and transaction costs are high, households with a more diverse production may have improved access to better-quality diets than those with a less varied and subsistence production. Commercialized households may have a higher likelihood of consuming diverse food than their subsistence counterparts (Jones et al., 2014; Sibhatu et al., 2015), with the likelihood potentially increasing with the level of market integration (Hirvonen & Hoddinott, 2017).

The paper is organized as follows: Sect. 2 provides details on the data and methods used; Sect. 3 presents the empirical results; Sect. 4 discusses the potential limitations of the study and concludes.

## Materials and methods

### Data and variables

Data for this study come from the 2016 Viet Nam Household Living Standards Survey (VHLSS) that covered a total of 46,995 households residing in 3,133 communes/wards, although consumption and production data were collected from a reduced, but still statistically representative sample of 9,399 households. VHLSS is a national consumption and expenditure survey representative at the national, regional, urban/rural, and provincial levels and was conducted through face-to-face interviews across all four quarters of 2016. Data for household level food consumption was collected in two parts – festive and recurrent. Festive food^3^ consumption was based on a 12-month recall, including events such as the Lunar New Year, Christmas and Independence Day. Recurrent food^4^ consumption was based on a 30-day recall, excluding events such as festive occasions, parties, weddings and funerals. Other socioeconomic, demographic, and geographical information was also collected and have been used to construct variables for the multivariate analyses.

The relatively long (30-day) recall period for recurrent food consumption might be a potential source of measurement bias. Some evidence suggests that longer recall periods are more likely to cause partial or complete item non-response among respondents (Sudman & Bradburn, 1973), and lower levels of consumption are reported with increasing recall periods (Beegle et al., 2012; FAO & World Bank, 2018; Grosh et al., 1995). The effect of different recall periods is also not uniform across food groups (Friedman, 2016). Unfortunately, due to data limitations we are unable to control for this potential bias. Additionally, the VHLSS 2016 did not collect information on the cooking method of each food item. However, existing evidence suggests that there is no significant difference in the nutritional value between raw and cooked food items when considering dietary energy, protein, fats, carbohydrates, total fiber and calcium (Moltedo et al., 2021). Hence, while we are unable to account for the cooking method in our analysis due to missing information in our data source, we do not expect it to significantly bias our results.

A nutrient analytic Food Conversion Table was developed by matching each food item in the VHLSS 2016 production and consumption modules with food composition data from the 2007 Vietnamese Food Composition Table (Ministry of Health & National Institute of Nutrition, 2007). Quantities in grams were adjusted for non-edible portions before converting them into nutrient equivalents. As proxies of agricultural potential and market access, we use precipitation and temperature data from the Copernicus Climate Change Service^5^ and travel time to the nearest market (by means of the most commonly available surface transport) from Weiss et al. (2018).

The reference period for agricultural production was the last 12 months. As such, agricultural production decisions and harvest occurred before household food consumption data were collected. We acknowledge that considering the dual role of households as both producers and consumers, household decisions about agricultural production, labour allocation and food consumption might have been interdependent, thereby introducing simultaneity between production and consumption analysed here.

### Dietary indicators

Seven complementary indicators are used to proxy the outcome variable of interest‒household dietary quality‒ based on recommendations for a healthy diet (INDDEX Project, 2018; World Health Organization, 2020). Three indicators measure daily consumption of three food groups and nutrients expressed in grams (g) per capita per day (computed by dividing total daily household consumption by household size) ‒F&V, dietary fiber, animal protein; one indicator measures per capita daily household dietary energy consumption (DEC) intake expressed in kilocalories (kcal); and three indicators measure whether the household has a balanced share of dietary energy from three macronutrients ‒carbohydrates, fats, and protein consumption. A healthy diet should consist of fruits, vegetables, legumes, nuts, and whole grains. High-fiber diet has several advantages including for bowel movements and health, cholesterol and sugar levels, and weight control. Fruits and vegetables are important sources of essential vitamins and minerals the body needs for survival and to say healthy. Diets with disproportionately low or high dietary energy intake from a given macronutrient should be avoided since that is a sign of underconsumption (disproportionately high share of carbohydrates) or overconsumption (disproportionately high share of lipids and sometimes proteins). According to WHO, the contribution of carbohydrates, fats, and protein in a healthy diet should range between 55–75%, 15–30%, and 10–15%, respectively (World Health Organization, 2003).

Viet Nam is a major world producer of rice (Nguyen, 2017), with this commodity featuring quite heavily in the Vietnamese diet. This strong reliance on rice may potentially increase the incidence of macronutrient imbalances. Additionally, the government has a target balanced diet with carbohydrate, fat, and protein contributions, respectively, of 68%, 18%, and 14% of total DEC (Ministry of Health, 2012). This target split amongst the macronutrients is close to the breakdown we are using in our statistical analysis.

### Multivariate analysis

Since our focus is on estimating the relationship between production systems and dietary quality, we estimate the model in Eq. 1 on a sub-sample of households producing agricultural output, using Ordinary Least Squares (OLS):

1 $${Y}_{i}={\alpha }_{o}+{\alpha }_{1}{Rice}_{i}+ {\alpha }_{2}{Other}_{i}+{\alpha }_{3}{Live}_{i}+{\alpha }_{4}{Aqua}_{i}+{\alpha }_{5}{F\&V}_{i}+{\alpha }_{6}{Live\_F\&V}_{i} +{\alpha }_{7}{Live\_Aqua}_{i}+{\alpha }_{8}{Aqua\_F\&V}_{i} + {\alpha }_{9}{VAC}_{i}+\mathbf{\rm B}\mathbf{^{\prime}}{{\varvec{X}}}_{i}+{\varvec{\Omega}}{{\varvec{Z}}}_{i}+{\epsilon }_{i}$$

where $i$ is an index for household; $Y$ is one of the four continuous dietary indicators (per capita F&V, fiber, animal protein consumption (g/day); per capita dietary energy consumption (kcal/day); $Rice$ is an indicator for whether the household produced *only* rice (omitted category); $Other$ is indicator for whether household produced any *other crops*^6^ (both food and non-food) and no other VAC component, with/without rice; *Live*^7^is indicator for whether the household produced livestock and no other VAC component; *Aqua*^8^ is indicator for whether the household produced aquaculture and no other VAC component; *F&V*^9^ is indicator for whether the household produced F&V and no other VAC component; $Live\_F\&V$ is indicator for whether the household produced livestock and F&V; $Live\_Aqua$ whether household produced livestock and aquaculture; $Aqua\_F\&V$ whether household produced aquaculture and F&V; $VAC$ is indicator for whether the household produced livestock, aquaculture, and F&V. All categories except the first two (*Rice* and *Other*) are defined to include households with or without rice or other crops. Indicators for the different production systems are defined such that the categories are mutually exclusive.

The matrix ${\varvec{X}}$ contains household-level socioeconomic conditioning variables, including household size (number of household members unadjusted for adult equivalent scales), area of residence (urban/rural), gender and education of the household head, asset-based household wealth index,^10^ total agricultural production value (in the past 12 months, measured in thousands of *Dong*), market access (measured as time taken in minutes to travel to the nearest market), and the percentage of household’s total food monetary value from purchases. The matrix ${\varvec{Z}}$ includes the historical mean and coefficient of variation of precipitation and temperature between 1995 and 2015, as they are likely to affect availability and accessibility of food. The model error term is expressed by ${\epsilon }_{i}$.

A logit model is estimated according to Eq. 2 for the three binary outcome variables measuring whether the household has a balanced dietary energy derived from carbohydrates, fats, and protein.

2 $$\mathrm{Pr}\left({Y}_{i}=1\right)=F({\beta }_{o}+{\beta }_{1}{Rice}_{i}+ {\beta }_{2}{Other}_{i}+{\beta }_{3}{Live}_{i}+{\beta }_{4}{Aqua}_{i}+{\beta }_{5}{F\&V}_{i}+{\beta }_{6}{Live\_F\&V}_{i} +{\beta }_{7}{Live\_Aqua}_{i}+{\beta }_{8}{Aqua\_F\&V}_{i} + {\beta }_{9}{VAC}_{i}+{\varvec{\Gamma}}\mathbf{^{\prime}}{{\varvec{X}}}_{i}+{\varvec{\Delta}}{{\varvec{Z}}}_{i})$$

where $F(z)={e}^{z}/(1+{e}^{z}$) is the cumulative logistic distribution and notation used is the same as in Eq. 1. All descriptive summaries and parameter estimates are weighted using survey weights, and standard errors have been clustered at the stratum level. Additionally, for the logistic specification, we are presenting the marginal effects.

Under this framework production and consumption decisions are taking place at different points in time and the associated data have been collected over different recall and reference periods. Production and consumption data have been collected based on different recall and reference periods; production data were referred to the 12 months prior to the interview, while consumption data were referred to the 12 months (for festive food consumption) and 30 days (for recurrent food consumption) prior to the interview. As such, production output occurred before consumption data collection, so it is reasonable to assume that production decisions have affected consumption outcomes. Furthermore, we are examining the link between production and dietary consumption, without analyzing nutritional outcomes that would be more prone to simultaneity bias.

## Results

### Descriptive

Table 1 shows the distribution of households by production system. Approximately 38% (almost 9.5 million) out of the almost 25 million households in Viet Nam are non-producers, i.e., do not have any agricultural output. A full VAC model of production, that is including all the three subcomponents (F&V, aquaculture and livestock), is implemented by only 14% of producers. Livestock with F&V, and livestock-only are the two most common VAC components (34% and 18% of producers, respectively).

**Table 1:** Distribution of households by production system (national level)

Production Category	Number of households	Frequency among producers (%)
**Non-Producers (does not produce rice, VAC crops or other crops)**	**9,418,948**	-
Rice only (produces only rice; no other crops and no VAC crops)	1,216,244	8
Other crops (no VAC crops, with/without rice)	768,391	5
Livestock (with/without rice/other crops)	2,768,769	18
Aquaculture (with/without rice/other crops)	649,817	4
F&V (with/without rice/other crops)	1,734,649	12
Livestock and F&V (with/without rice/other crops)	5,207,048	34
Livestock and Aquaculture (with/without rice/other crops)	495,102	3
Aquaculture and F&V (with/without rice/other crops)	339,753	2
VAC (with/without rice/other crops)	2,113,431	14
**Total producers**	**15,293,204**	**100**
**Total producers + non-producers**	**24,712,152**	**-**

Table 2 panel A presents a descriptive summary of the full sample and separately for non-producers and producers, along with significance levels from tests of equality of means across the two groups. Average household size is 3.7 members, average household head is over 50 years of age, female-headed households are about a quarter of the total, average residential area covers about 80 square meters, a third of the sample resides in urban areas, and average travel time to the nearest market is approximately 35 min. Producers are more likely to live in larger households, be male-headed, and travel longer to the nearest market, while they are less likely to fall in the upper education levels or to reside in urban areas. Finally, we observe a significant difference in the average wealth status between producers and non-producers, much lower for the former.

**Table 2:** Descriptive summary

Variable	Full Sample	Non-Producers	Producers
*Number of households in VHLSS 2016 (unweighted)*	9,399	3,209	6,190
	Mean	Mean	Mean
(A)Independent Variables			
*Socio-economic*			
Household size	3.74	3.49	3.90***
Household head age	52.09	53.16	51.44***
Female household head (%)	25.75	36.80	18.95***
Mode education level in the household (%)^a^			
None	15.64	14.45	18.12***
Primary	23.27	19.33	26.7***
Lower Secondary	28.68	21.17	32.28***
Higher Secondary	20.27	23.76	17.1***
College and above	12.13	21.29	5.81***
Urban (%)	32.05	63.54	12.76***
Household residential area (m^2)	79.7	82.07	78.24***
Household Wealth Index (0–1)	0	0.36	-0.22***
Nearest Market (minutes)	34.77	23.62	41.64***
*Agriculture*			
Total agricultural land (hectares)	2.95	0	4.76***
Number of food groups produced^b^	1.75	0	2.82***
Number of food items produced	3.14	0	5.07***
Household produces livestock (%)	42.83	0	69.66***
Household produces fish or shrimp (%)	14.56	0	24.54***
Household produces fruits or vegetables (%)	38.02	0	62.34***
Total production value (‘000 *dong*)	16154.4	0	26103.74***
*Assets and income*			
Number of different kinds of durable goods	11.84	12.93	11.17***
Total expenditure per capita (‘000 *dong*)	37603.44	53987.26	27512.79***
*Biophysical (1995*–*2015)*			
Mean annual temperature (degree Celsius)	24.96	25.69	24.52***
Temperature coefficient of variation (CV)	0.13	0.10	0.14***
Mean monthly precipitation (mm)	163.17	164.14	162.57***
Precipitation CV	0.79	0.78	0.80***
(B)Dependent Variables			
Fruits & Vegetable consumption (g/day/per capita)	76.39	87.70	69.42***
Fiber consumption (g/day/per capita)	3	3	3
Protein from animal origin (g/day/per capita)	19.88	20.99	19.20***
Dietary energy consumption (kcal/day/per capita)	2,483.67	2,546.13	2,440.77***
Proportion of calories from carbohydrates (%)^c^	68.22	66.64	69.71***
Proportion of calories from protein (%)	12.89	13.39	12.41***
Proportion of calories from fats (%)	16.81	17.95	15.64***

All values, unless mentioned otherwise, are weighted using VHLSS2016 sampling weights, ensuring national representativeness. Stars indicate statistical significance of the test of equality of means between Producers and Non-Producers

* p < 0.1; ** p < 0.05; *** p < 0.01

^a^The mode education level in the household refers to the attained level of education prevalent amongst the highest number of household members

^b^Food groups are the following: (1) rice, (2) staple food crops, non-staple food crops and other annual crops, (3) annual and perennial industrial crops, (4) fruit trees, (5) animal husbandry, hunting, trapping and domesticated birds, (6) fishery

^c^The three proportions (from carbohydrates, protein and fats) do not add up to 100%, because proportion of calories derived from fiber and alcohol have not been shown (together they account for approximately 2% of dietary energy consumption)

An average Vietnamese household produces about 1.8 of the six food groups considered and slightly more than three unique food items, with 43%, 38%, and 9% of households producing livestock, F&V, and fish or shrimp, respectively. Total annual production value is estimated at 19.6 million *dong* (equivalent to approximately USD$844 in June 2020). On average, a household owns about 12 unique durable assets, and has a total per capita expenditure above 37.6 million *don*g; noting the statistically significant differences, we can indicate that producers are worse off than non-producers along several socio-economic dimensions.

Viet Nam is divided into the highlands and the Red River Delta in the north, and the Central Mountains, the coastal lowlands in the central region, and the Mekong River Delta in the south. A dip in elevation from the northwest to the southeast exists, along the two major rivers: The Red River and Mekong River. They flow in the northern and southern regions, respectively, being an important source of soil and nutrients for agricultural land. Arable land is however limited, being only 20% of total land. Soils are quite diverse in the country: the three main soil groups are mountainous, hilly, and delta soils. Mountainous and hilly soils tend to be acidic, degrade quickly, and exhibit poor fertility. In contrast, soils in delta regions are primarily alluvial, highly fertile, and are suited for extensive cultivation (Abidoye et al., 2017). Average annual temperature in the country was approximately 25 degrees Celsius during the 20-year period 1995–2015, with an average monthly precipitation of approximately 163 mm. Variability of precipitation is greater than temperature’s although, given the shape of the country, variability varies quite substantially according to the diverse agro-climatic zones. As with socioeconomic variables, temperature and precipitation are also found to statistically differ by producers and non-producers, indicating that the two groups reside in different areas of the country. This is unsurprising, as we would expect producer households to live in higher agricultural-potential regions than non-producers, the latter living predominantly in urban areas.

Table 2 panel B shows average values of dietary indicators across the two household groups. Average F&V consumption in Viet Nam is approximately 70 g per day/capita,^11^ while protein consumption from ASFs averages slightly less than 20 g per day per capita. The share of dietary energy (kcal) obtained from carbohydrates, protein, and fats averaged approximately 69%, 13%, and 16%, respectively. Significant differences in the dietary patterns of producers and non-producers can be found. Producers show a lower dietary energy consumption than non-producers, likely due to a greater reliance of the latter on processed food, which are usually high in sugars and fats and frequently consumed away from home. Non-producers also derive a higher share of their dietary energy from protein and fats, pointing towards their relatively higher economic status.

**Fig. 1:**
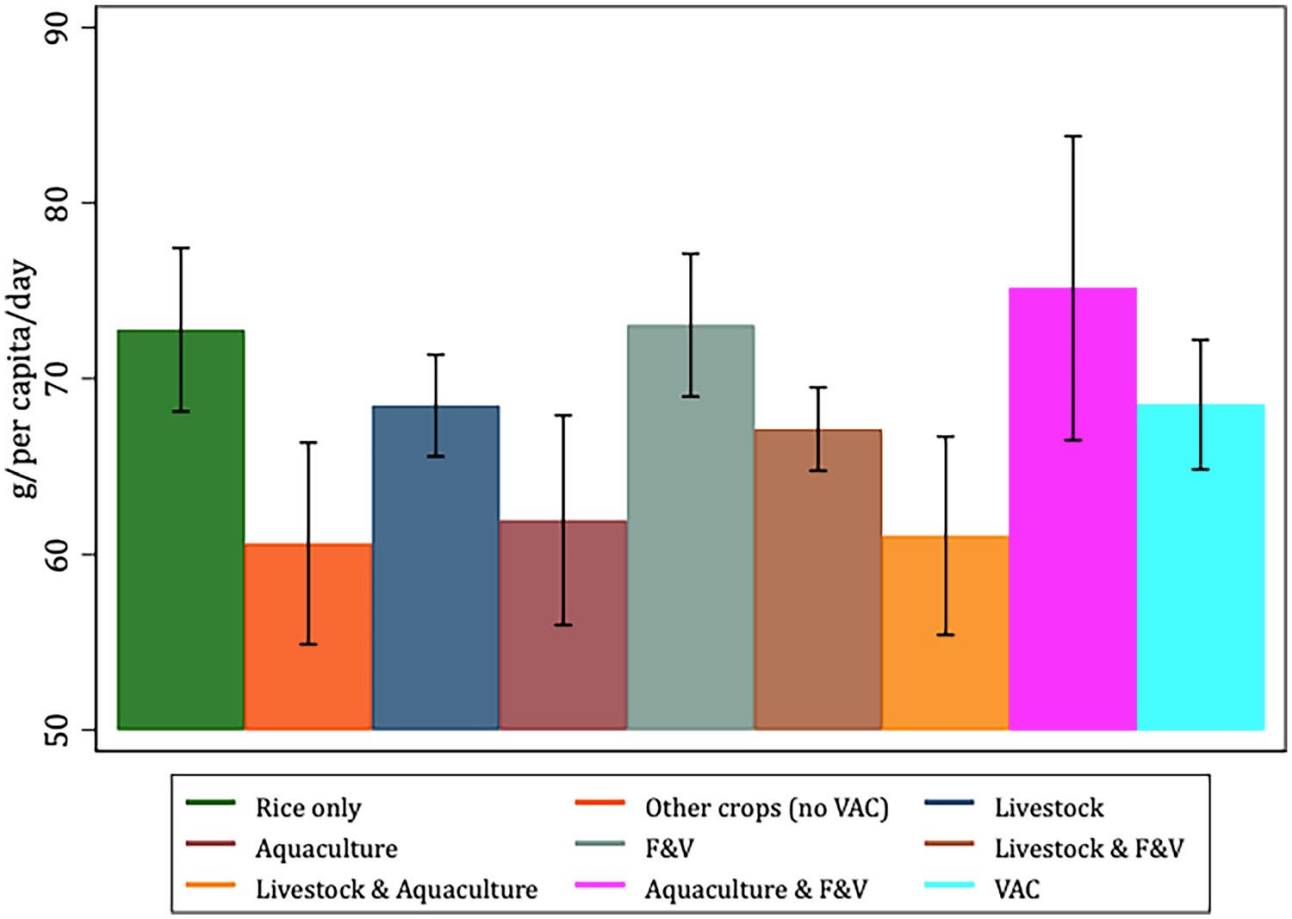
Fruit and Vegetable consumption, by production category

**Fig. 2:**
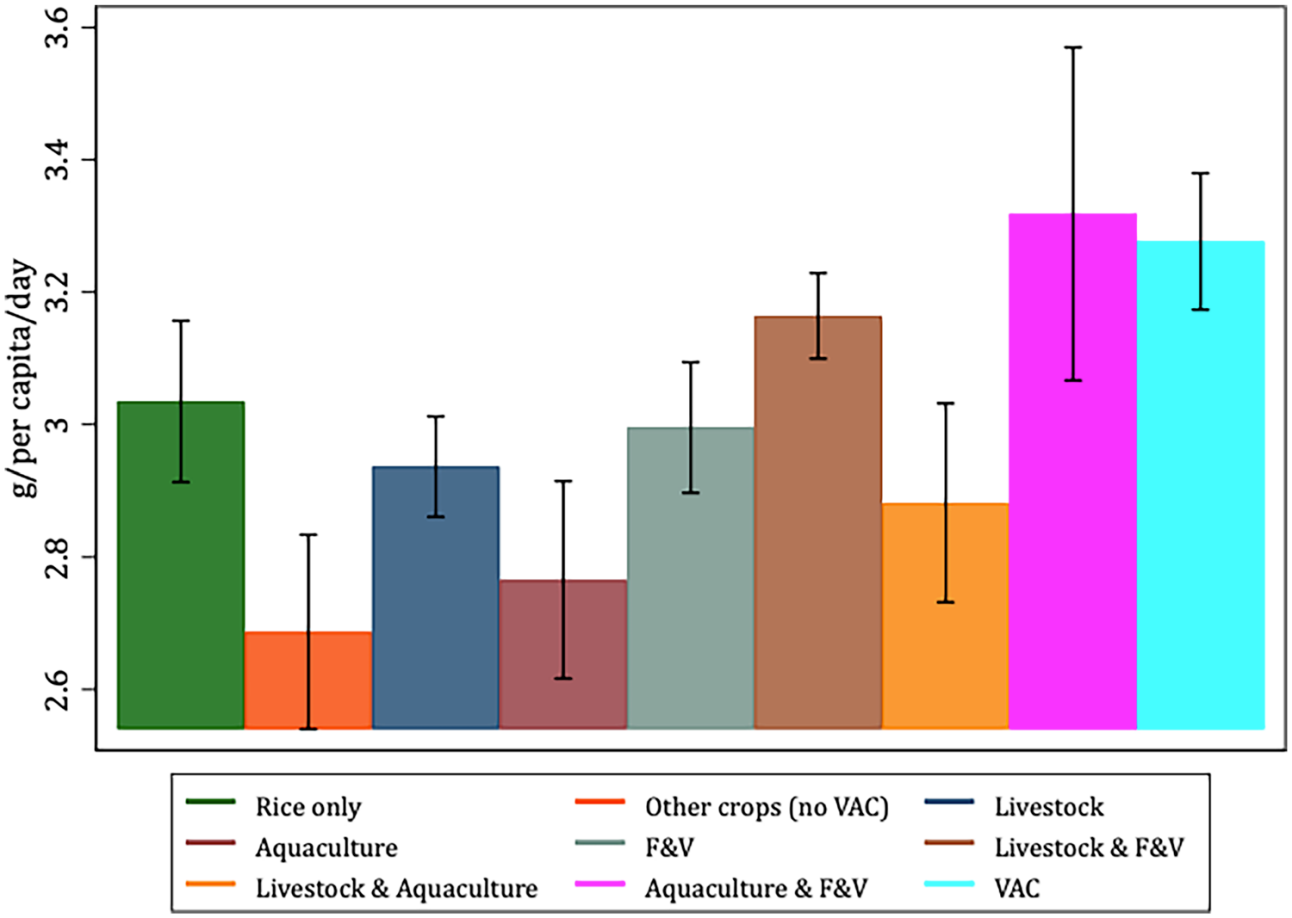
Fiber consumption, by production category

**Fig. 3:**
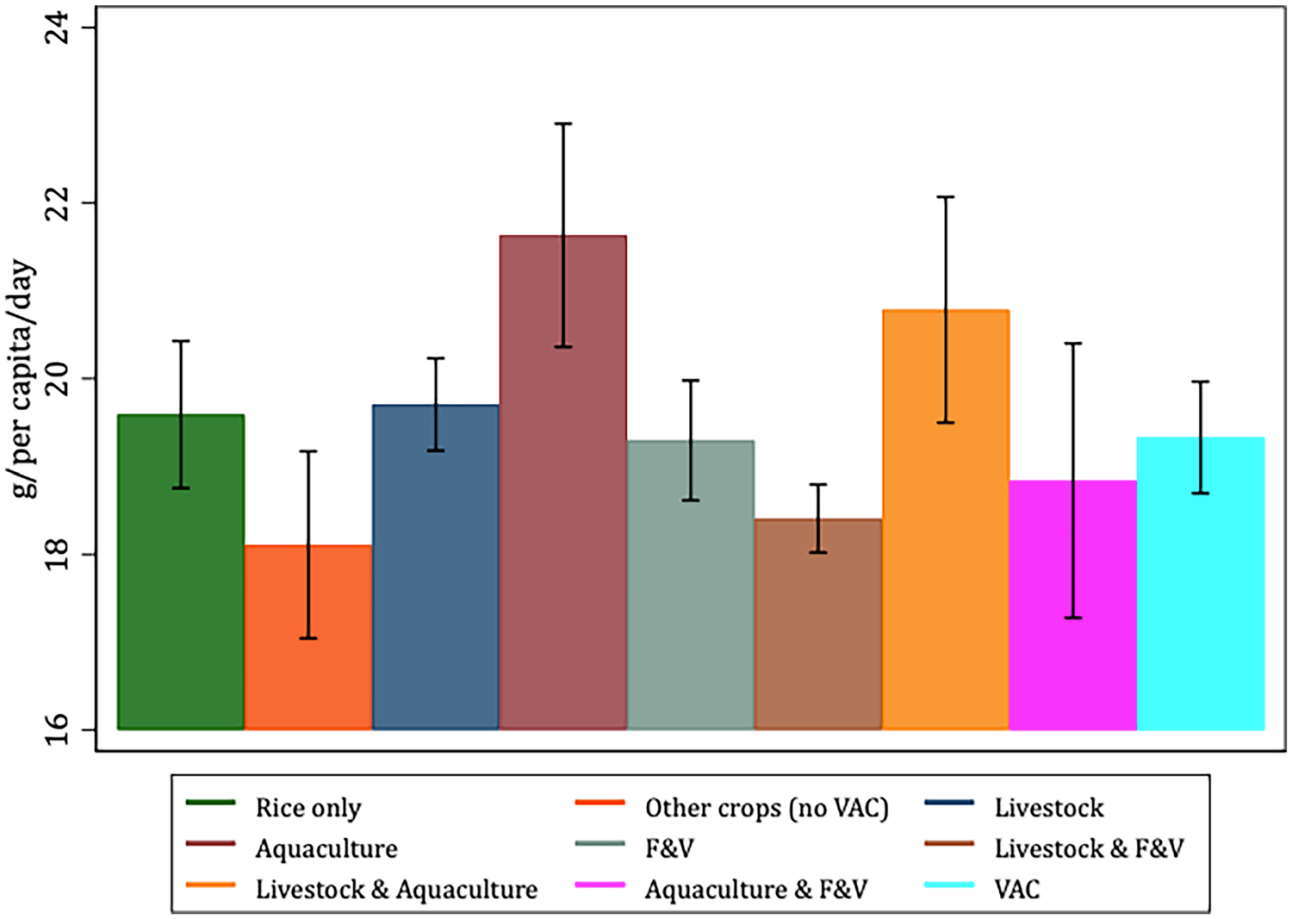
Animal protein consumption, by production category

**Fig. 4:**
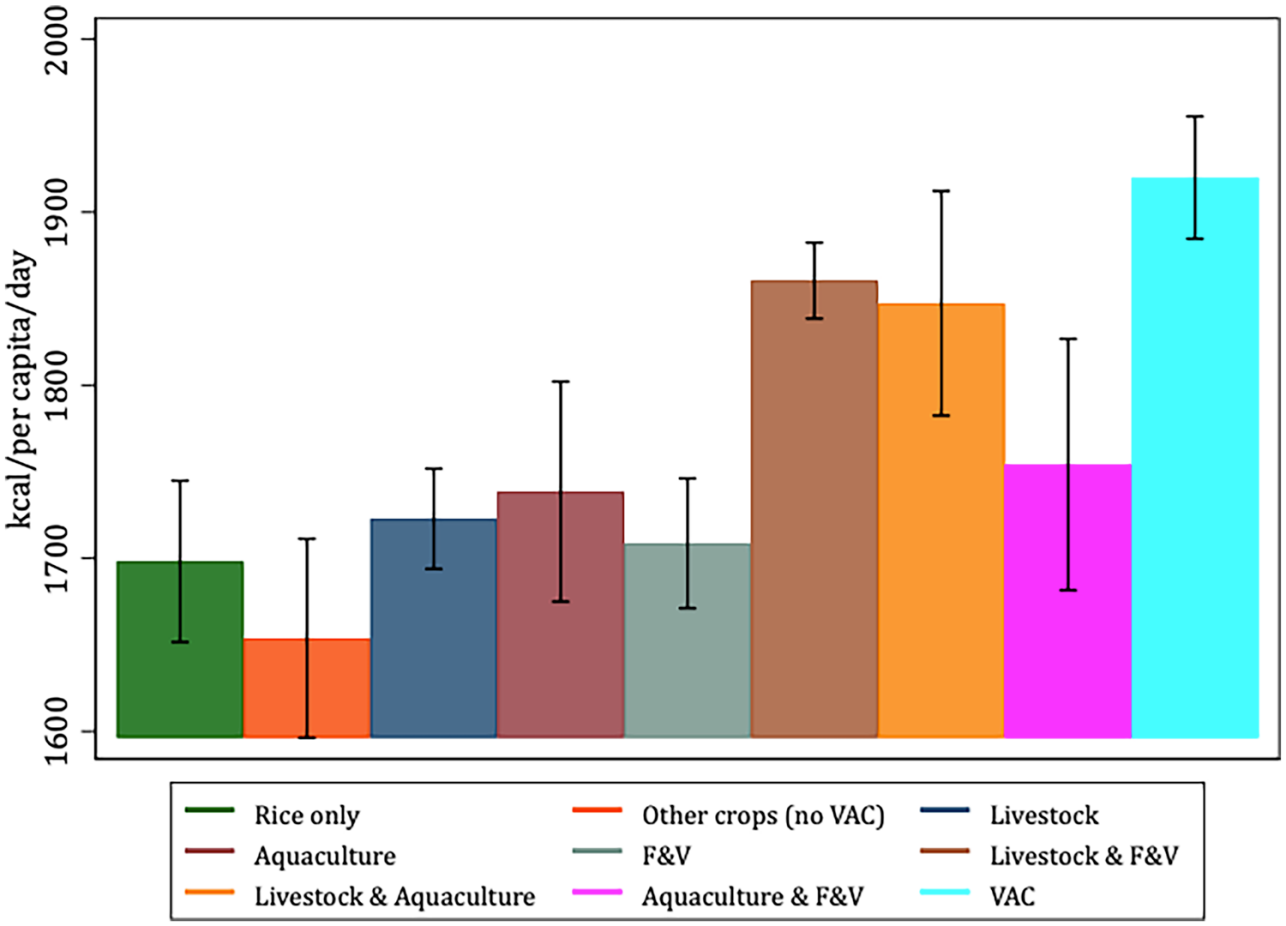
Dietary energy consumption, by production category

**Fig. 5:**
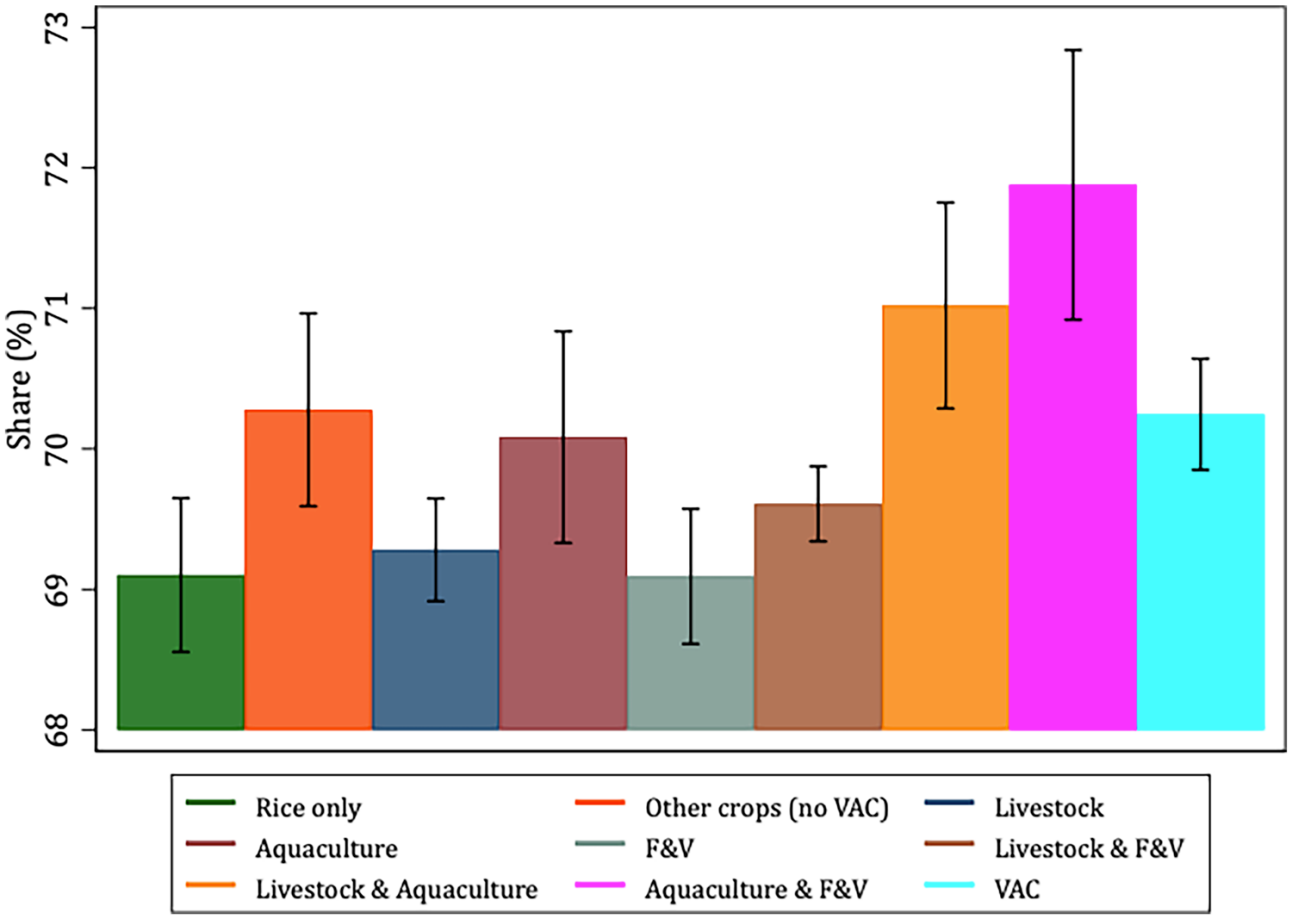
Share of dietary energy from carbohydrates (excluding fiber), by production category

**Fig. 6:**
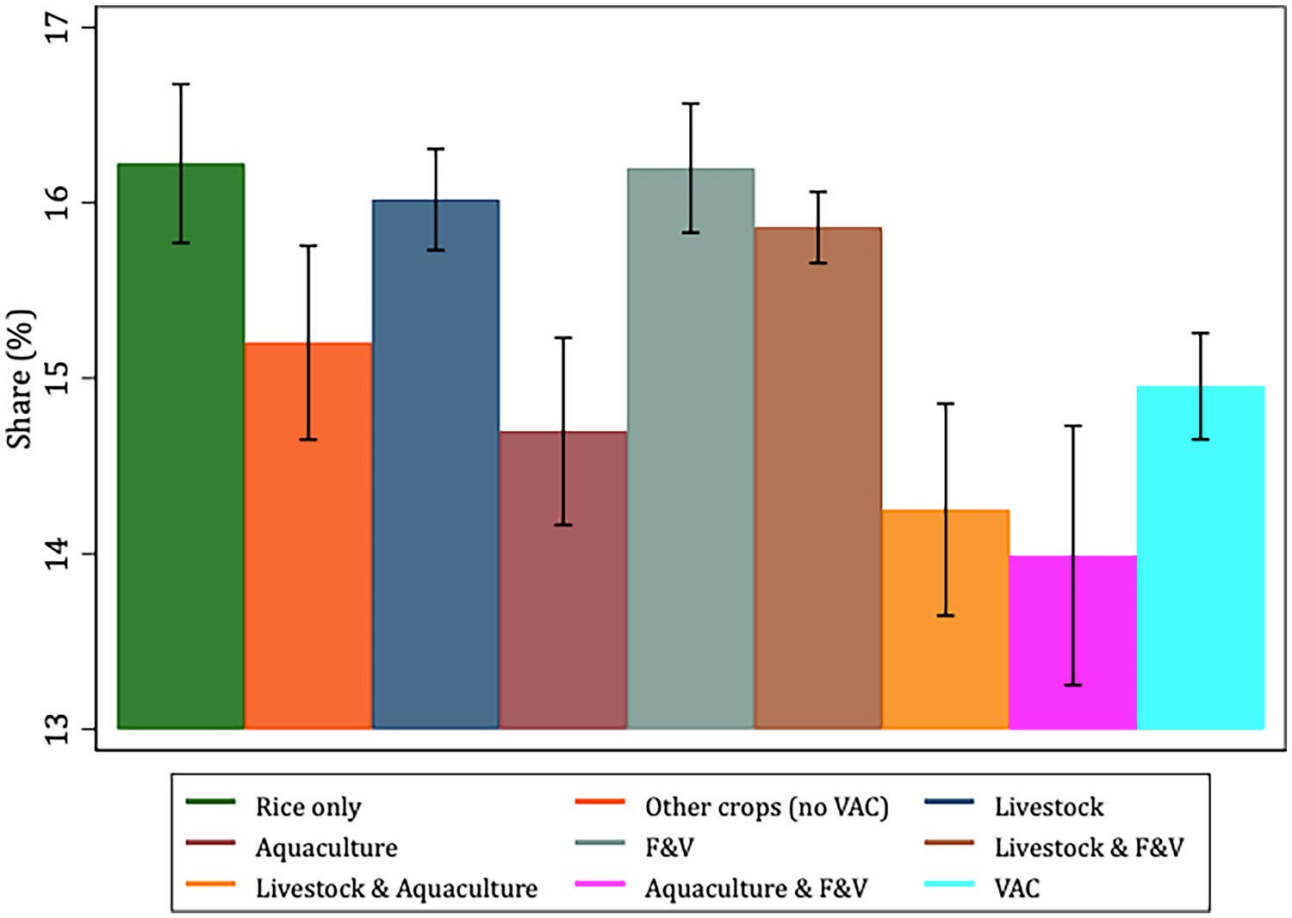
Share of dietary energy from fats, by production category

To better characterize and justify the use of the VAC categories *vis-à-vis* our selected variables of interest, Figs. 1, 2, 3, 4, 5 and 6 present the bivariate relationship between the production categories and our outcome variables.

The highest daily per-capita F&V consumption is among rice-only, F&V and aquaculture & F&V producers (Fig. 1).

Fiber consumption (Fig. 2) is highest among livestock & F&V, aquaculture & F&V and VAC producers. This finding is in line with expectations as fruits and vegetables are a significant source of dietary fiber (Dhingra et al., 2012). Interestingly, rice-only producers consume a relatively similar amount of fiber as F&V producers. Producers of other crops on average have the lowest fiber consumption, hence also lower than for VAC producers.

Two production groups, aquaculture and livestock and aquaculture have the highest per capita animal protein consumption (Fig. 3). This is in line with our expectations since these households would be able to complement their animal source food intake from their own production. Looking at the different production categories, households derive a greater amount of animal protein intake from aquaculture (and its related production categories) than from livestock production. This could have implications on which components of the VAC system is most efficient in improving household diets.

Figure 4 shows that VAC, livestock, F&V, and livestock and aqua producers are associated to the highest dietary energy consumption (DEC). In contrast, rice-only producers have the lowest DEC.

Livestock and aquaculture and aquaculture and F&V producers derive a relatively larger portion of their dietary energy from carbohydrates (Fig. 5), and this finding is not surprising as these two producer categories are among the poorer groups and might afford mostly carbohydrates given they are often the cheapest source of dietary energy.

**Fig. 7:**
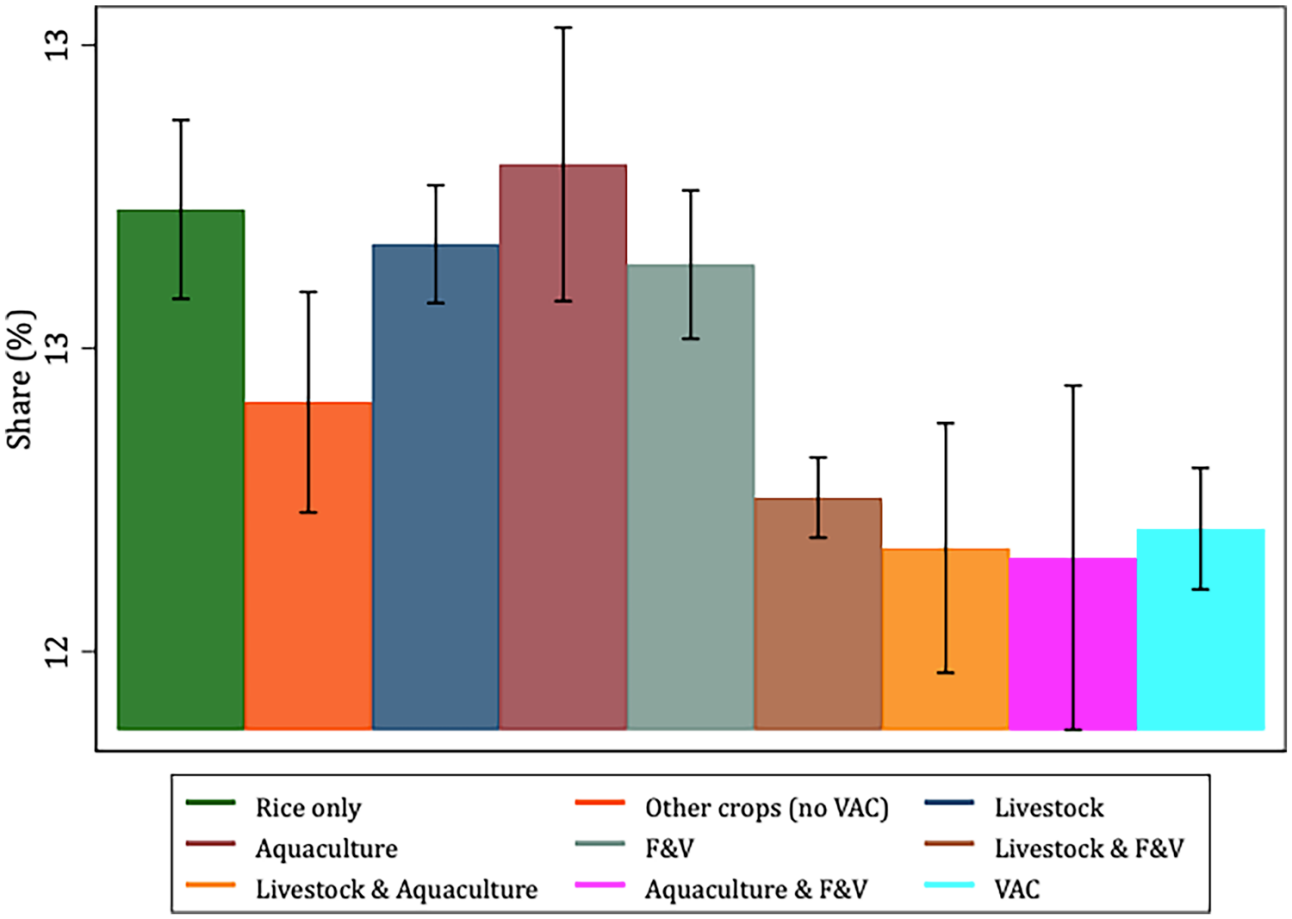
Share of dietary energy from proteins, by production category

Figure 6 shows that VAC producers derive a significantly lower share of dietary energy from fats compared to rice-only, livestock, F&V and livestock & F&V producers. Also, rice-only, livestock, aquaculture and F&V producers derive the highest share of dietary energy from proteins (Fig. 7).

Table 3 presents the averages of some selected household-level indicators across production categories -including VAC-, where statistical significance is related to the average value associated to a particular production category being different from the rest of the production categories taken together. For example, household size of VAC producers is significantly higher from the rest of other production categories. Similarly, VAC households seem to be statistically different than the other production categories put together along other socio-economic dimensions such as household wealth, travel distance to the nearest market, size of agricultural land and share of food consumption sources from purchases. These findings suggest that VAC households might systemically be different from other producers also along other dimensions, namely dietary habits, something we will indeed explore in the econometric model presented below.

**Table 3:** Summary of production categories across household characteristics

Household characteristic (mean)	Rice only (produces only rice; no other crops and no VAC crops)	Other crops (no VAC crops, with/without rice)	Livestock (with/without rice/other crops)	Aquaculture (with/without rice/other crops)	F&V (with/without rice/other crops)	Livestock and F&V (with/without rice/other crops)	Livestock and Aquaculture (with/without rice/other crops)	Aquaculture and F&V (with/without rice/other crops)	VAC (with/without rice/other crops)
Household size	3.81*	3.93	3.86*	4.07	3.72***	3.94	4.09	3.86	4.16***
Household Wealth Index	-0.14***	-0.23	-0.09***	-0.39***	-0.09***	-0.35***	-0.32	-0.44***	-0.36***
Market access (minutes)	31.10***	49.61**	41.87***	30.21***	38.00***	54.31***	32.03***	32.01***	51.20***
Agricultural land size (Hectares)	6.76***	7.95***	3.56***	1.86***	3.61***	5.25	5.48	3.95	6.52***
Share of food consumption from purchases (%)	96***	98***	91***	93***	90***	80***	88	90	76***

Stars indicate statistical significance of average values associated to a particular production category being different from the rest of the production categories taken together

* p < 0.1; ** p < 0.05; *** p < 0.01

**Fig. 8:**
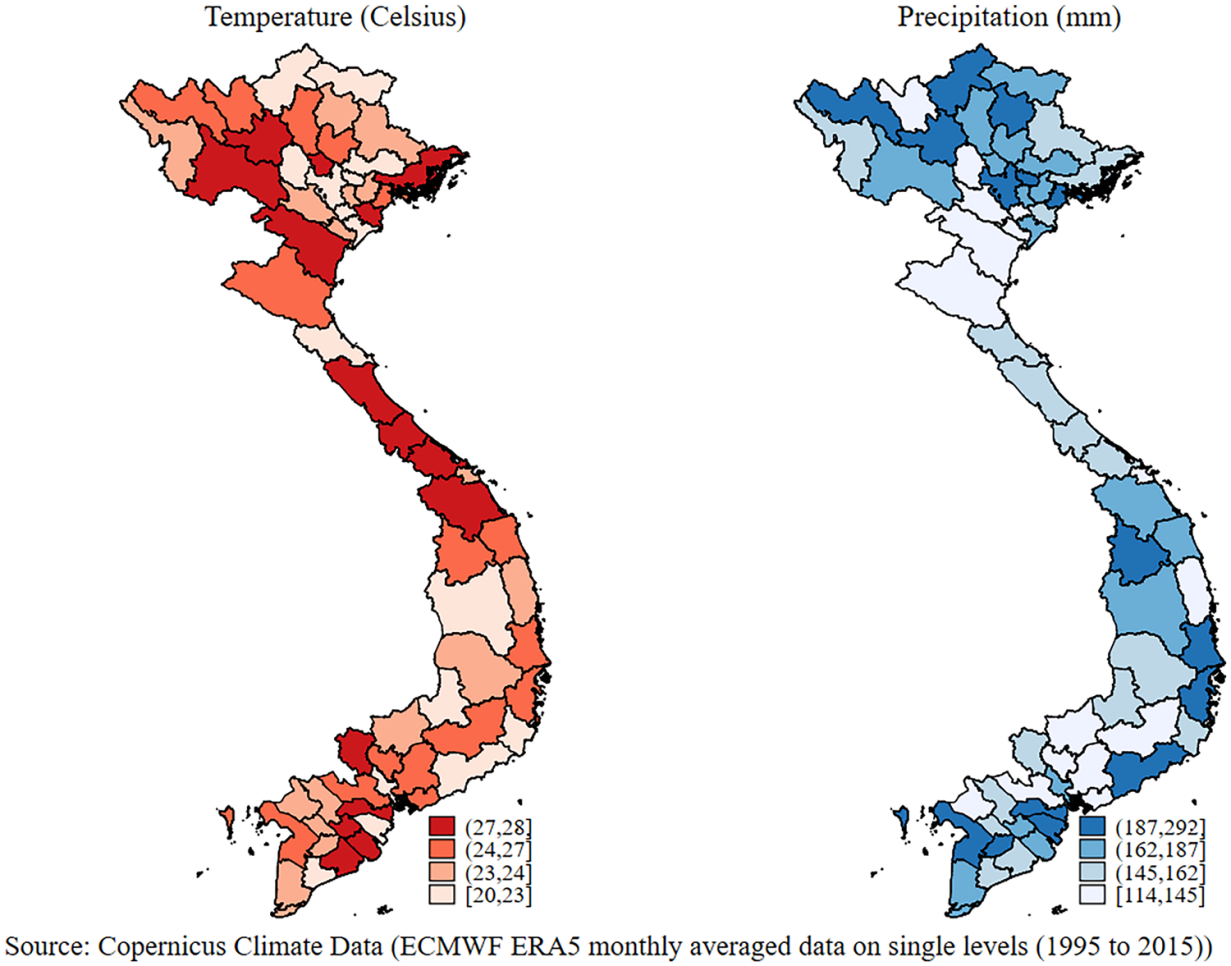
Spatial variation of temperature and precipitation (average by province)

The spatial variation of temperature and precipitation (Fig. 8) signals that climate is highly diverse across the country, with three broad regions—humid and subtropical in the north, tropical monsoon in the center, and tropical savannah in the south. High levels of humidity prevail throughout the year, ranging between 84 and 100 per cent. Monthly precipitation ranges from 114 to 292 mm. Average temperatures range between 21 and 27 degrees Celsius and are higher in the southern areas of the country. The southern regions show both the highest average temperature and highest rainfall. More generally, there is substantial variation across regions and provinces in both rainfall and temperature. Biophysical variables are strongly spatially correlated to different household production activities and dietary outcomes, providing justification to control for these biophysical variables in our multivariate framework.

### Econometric

Parameter estimates of the multivariate regression model underlying Eq. 1 using OLS are presented in Table 4.

**Table 4:** OLS Estimation

Independent Variables	Fruits and Vegetable Consumption	Fiber Consumption	Animal Protein Consumption	Dietary Energy Consumption
Household size	-9.775***	-0.255***	-2.218***	-154.9***
	[-19.65]	[-17.80]	[-26.33]	[-23.44]
Female household head	0.0440	-0.0760	0.510	-34.31
	[0.02]	[-1.58]	[1.60]	[-1.48]
Rural	-3.178	0.0137	-0.0563	85.51***
	[-1.41]	[0.23]	[-0.14]	[2.89]
*Most common educational qualification*				
Primary	4.762**	0.114**	-0.269	42.88
	[2.51]	[2.00]	[-0.77]	[1.56]
Lower Secondary	5.703***	0.110*	0.174	70.83**
	[2.96]	[1.93]	[0.50]	[2.55]
Higher Secondary	0.236	-0.0396	0.0272	131.8***
	[0.10]	[-0.63]	[0.07]	[3.94]
College & above	6.919*	0.155*	0.170	79.74
	[1.94]	[1.73]	[0.29]	[1.64]
Household wealth index	12.23***	0.163***	3.343***	191.5***
	[11.22]	[4.98]	[17.69]	[12.39]
Total production value (millions of *dong*)	0.0424	0.00292***	0.0331***	2.318***
	[1.47]	[3.71]	[6.38]	[6.32]
*Household produces*				
Other crops (no VAC; with/without rice)	-4.133	-0.143	-1.530**	-114.6**
	[-0.97]	[-1.26]	[-2.07]	[-1.97]
Livestock (with/without rice/other)	-1.790	0.00784	-0.397	-13.03
	[-0.57]	[0.10]	[-0.73]	[-0.30]
Aquaculture (with/without rice/other)	3.290	-0.0343	2.401***	19.21
	[0.70]	[-0.28]	[2.96]	[0.32]
F&V (with/without rice/other)	0.196	-0.0355	-1.227**	-63.63
	[0.06]	[-0.39]	[-2.06]	[-1.36]
Livestock and F&V (with/without rice/other)	0.900	0.166**	-0.538	-12.64
	[0.28]	[2.04]	[-0.97]	[-0.30]
Livestock and Aquaculture (with/without rice/other)	-5.231	-0.0878	0.778	31.50
	[-1.41]	[-0.86]	[0.97]	[0.49]
Aquaculture and F&V (with/without rice/other)	8.428*	0.352**	-1.273	-41.29
	[1.71]	[2.33]	[-1.46]	[-0.58]
VAC (with/without rice/other)	3.940	0.291***	0.141	12.34
	[1.14]	[3.14]	[0.23]	[0.27]
Travel time to nearest market (minutes)	-0.211***	-0.000215	-0.0182***	1.911***
	[-5.40]	[-0.20]	[-2.77]	[3.61]
Ratio of purchased food to total food value	1.485	-0.0169	0.458	52.29**
	[0.70]	[-0.29]	[1.48]	[2.11]
*Biophysical (1995*–*2015)*				
Mean Temperature (monthly averages, Celsius)	-4.355***	-0.0709**	-0.912***	-31.08**
	[-3.70]	[-2.28]	[-4.71]	[-2.02]
C.V. Temperature	-91.38***	-0.995	-23.85***	-1638.3***
	[-2.85]	[-1.06]	[-3.95]	[-3.50]
Mean Precipitation (monthly averages, mm)	0.0572	-0.00199**	0.0108*	-0.206
	[1.60]	[-2.23]	[1.90]	[-0.52]
C.V. Precipitation	32.45**	-1.318***	2.823	-376.5*
	[2.18]	[-2.62]	[1.19]	[-1.88]
Constant	212.3***	7.382***	50.38***	4121.4***
	[6.59]	[8.93]	[9.17]	[9.24]
F-Statistic	30.36	22.24	44.31	28.48
F-Statistic P-value	8.12e-136	1.80e-99	2.54e-193	1.35e-127
R-square	0.169	0.107	0.213	0.146
Observations	6147	6147	6147	6147

*t* statistics presented in brackets

* p < 0.1; ** p < 0.05; *** p < 0.01

Larger households seem to have worse diets using the different continuous dietary indicators; for one additional household member, consumption of F&V, fiber, animal protein, and dietary energy per-capita/day reduces by 9.8 g, 0.26 g, 2.2 g, and 155 kcal, respectively. Sex of household head does not bear any significant association with dietary energy consumption, while there is a positive correlation between residing in a rural location and the amount of dietary energy consumed, associated with an increase of 86 kcal/per capita/day. Compared to the base level of no education, higher education levels are significantly positively correlated across the indicators considered. However, this trend is not uniform, with the higher secondary education level having a significant correlation only for dietary energy and not the other indicators. Household wealth is highly (1% level) significant and positively correlated with all dietary indicators, suggesting that wealthier households can benefit of access to higher quality food, unsurprisingly. Total production value (measured in millions *dong*) is significantly positively correlated with all the three outcome variables, with a unit increase being associated with very small increases of 0.003 fiber g/per capita/day, 0.03 animal protein g/per capita/day, and 2.3 dietary energy consumption kcal/per capita/day. The limited magnitude suggests that while adoption of different production systems is indeed associated to different dietary outcomes, the size of the correlation is surprisingly lower that the one associated to other variables in the model.

Households producing *other crops* (and no VAC component) consume lower animal protein (by 1.5 g/per capita/day) and dietary energy (by 115 kcal/per capita/day) relative to rice-only producing households, our reference category. The relatively lower dietary energy consumption can be explained by the fact that specialized rice producers would have greater rice consumption, which is relatively high-energy dense. While production of F&V has a negative correlation with consumption of animal protein (by 1.2 g/per capita/day), aquaculture production is significantly positively correlated with animal protein (by 2.4 g/per capita/day), mostly due to fish and shrimp. Production of livestock alone or in conjunction with aquaculture or F&V does not seem to be significantly associated with ASF consumption; highlighting that it is not an important predictor relative to the other variables in the model.

Since F&V provide dietary fiber, households producing F&V and livestock, and those producing F&V and aquaculture are slightly more likely to consume fiber (0.17 and 0.35 g/per capita/day, respectively), relative to rice-only producers. However, the latter also report a relatively high consumption of F&V, as shown in Fig. 1.

Adoption of all three VAC components; aquaculture, livestock, *and* F&V, is significantly positively correlated only with fiber consumption (by 0.29 g/per capita/day). While parameter estimates associated to the other dietary indicators are positive, they are not statistically significant, and so adoption of VAC is not an important predictor relative to the other variables in the model. Full application of the VAC production system *might* improve nutrition. In fact, some VAC components do show significant positive associations -e.g., aquaculture and F&V with both F&V and fiber consumption; livestock and F&V with F&V and fiber consumption; both aquaculture and F&V with ASF consumption.

Market access, measured as the amount of time necessary to travel to the nearest market, is found to be significantly negatively correlated with F&V consumption (by 0.21 g/per capita/day) and animal protein consumption (by 0.02 g/per capita/day), but positively correlated with dietary energy consumption, though with negligible elasticity (by 1.9 kcal/per capita/day), indicating that households residing in remote areas might be just slightly disadvantaged in accessing nutritious food and hence tend to consume more staples.

Parameter estimates of indicators for adequacy of macronutrients consumption are reported in Table 5. An increase in household size significantly reduces the probability of households accessing a balanced diet in terms of macronutrients, with a reduction by 4.8% and 6.0% carbohydrates (excluding fiber and alcohol) and fats, respectively. Unlike the dietary indicators where there was no statistically significant correlation with female household headship, here we find that female-headed households are 4.6% more likely to achieve the recommended proportion of dietary energy from fats, potentially indicating that women might make better dietary choices when they are the main decision-makers in their household, supporting previous research (Amugsi et al., 2016; Rogers, 1996). For carbohydrates, the direction of the correlation between education and wealth are surprisingly opposite. While education level is significantly negatively correlated with access to recommended proportions of dietary energy from carbohydrates -where the reference category is no literacy-, household wealth is found to be significantly positively correlated with balanced shares of both carbohydrates and fats.

**Table 5:** Logistic Estimation

Independent Variables	Balanced DEC: Carbohydrates (excluding fiber)	Balanced DEC: Fats	Balanced DEC: Protein
Household size	-0.0480***	-0.0597***	0.00338
	[-12.93]	[-13.00]	[1.01]
Female household head	-0.0172	0.0455***	-0.00884
	[-1.25]	[2.80]	[-0.80]
Rural	-0.0156	-0.0495**	0.0190
	[-0.77]	[-2.25]	[1.44]
*Mode education level in the household (%)*			
Primary	-0.0342**	-0.0259	0.0285**
	[-2.40]	[-1.45]	[2.16]
Lower Secondary	-0.00482	-0.0288	0.0296**
	[-0.32]	[-1.52]	[2.22]
Higher Secondary	-0.0213	-0.0253	0.0228
	[-1.15]	[-1.15]	[1.44]
College & above	-0.00266	0.0236	0.00366
	[-0.09]	[0.75]	[0.16]
Household wealth index	0.118***	0.142***	0.00852
	[12.58]	[13.81]	[1.07]
Total production value (millions of *dong*)	0.000296	0.0000867	0.0000933
	[1.29]	[0.34]	[0.46]
*Production Categories*			
Other crops (no VAC; with/without rice)	0.0100	-0.00959	0.0272
	[0.32]	[-0.26]	[1.05]
Livestock (with/without rice/other)	0.0119	-0.0155	0.0183
	[0.53]	[-0.58]	[0.90]
Aquaculture (fish or shrimp) (with/without rice/other)	0.0109	0.0416	-0.0632*
	[0.37]	[1.08]	[-1.91]
F&V (with/without rice/other)	-0.0135	0.00183	0.0120
	[-0.53]	[0.06]	[0.55]
Livestock and F&V (with/without rice/other)	-0.0113	-0.0139	0.0320
	[-0.50]	[-0.54]	[1.64]
Livestock and Aquaculture (with/without rice/other)	0.0357	-0.0314	0.00777
	[1.19]	[-0.80]	[0.26]
Aquaculture and F&V (with/without rice/other)	-0.0511	-0.120**	0.0118
	[-1.24]	[-2.45]	[0.37]
VAC (with/without rice/other)	0.00114	-0.0478	0.0461**
	[0.04]	[-1.62]	[2.19]
Travel time to nearest market (minutes)	-0.000530*	-0.00150***	-0.000564**
	[-1.89]	[-4.33]	[-2.55]
Ratio of purchased food value to total food value	0.00262	0.0272	0.0282**
	[0.18]	[1.44]	[2.31]
*Biophysical (1995*–*2015)*			
Mean Temperature (monthly averages, Celsius)	-0.0220***	-0.0234**	0.0123*
	[-2.72]	[-2.20]	[1.73]
CV Temperature	-0.271	0.674**	0.0615
	[-1.08]	[2.11]	[0.26]
Mean Precipitation (monthly averages, mm)	-0.000242	0.000721**	-0.000195
	[-1.01]	[2.38]	[-0.98]
CV Precipitation	-0.252**	-0.0338	-0.0183
	[-2.32]	[-0.26]	[-0.21]
Observations	6147	6147	6147

*t* statistics presented in brackets

* p < 0.1; ** p < 0.05; *** p < 0.01

Unlike the previous OLS regression estimates, where we find some positive correlations between production categories and outcome variables, the logistic estimates on balanced proportions report mostly non-statistically significant associations. However, a significant correlation between VAC adoption and balanced share of protein exists. As found before, the separate VAC components show differential associations: producers of aquaculture are less likely to attain balanced protein consumption (-6.3%), and this negative association is almost doubled (+12%) for aquaculture and F&V producers when looking at balanced proportion in fat consumption.

We find a significantly positive probability (2.8%) between the share of food consumption expenditure from purchases and access to a balanced share of dietary energy from protein. Since rice is a relatively low source of protein, and a high prevalence of producers in the country are rice-producers (63%), this finding could indicate that relatively high protein-dense food items (e.g., animal source foods, legumes) are primarily sourced from markets, and this hypothesis is supported by the negative correlation between market access and all three macronutrient shares considered.

It is important to highlight the limitations of our study. Our data show that only 8.5% of the total sample and 13.8% of the sample of producers are VAC adopters, respectively. This relatively small proportion of VAC producers might render the associations observed quite weak, given the low sample size. Furthermore, while through this study we are unable to find clear dietary benefits due to the VAC integrated production system application relative to the other variables in the model, this is by no means a causal analysis from which direct impacts can be inferred. Much of the efficacy (or lack thereof) of the VAC system can potentially be explained by the fact that households engaged in the different production practices might simply systematically be different one another, and they can even self-select themselves in each production system. This finding is further supported by the positive associations of individual or combined components of the VAC production system with dietary outcomes. In other words, using cross-section observational data, our study cannot establish a rigorous causal mechanism between adoption of different production systems and dietary outcomes, something that future research should pursue taking advantage of experimental data.

## Conclusion

This study explored the relationship between consumption of various dietary components amongst households in Viet Nam in relation to the VAC production system. This system has been promoted by the Government since the 80 s, although to our knowledge no rigorous empirical assessment of its effects on nutrition exists. The objective of our paper is thus to examine the existence and extent of any linkages between VAC production system and diets.

While VAC adoption might be effective in regenerating soil quality and consequently improving environmental outcomes, our analysis finds that the relationship with nutritional outcomes is ambiguous. While we do find positive correlations between VAC production and some of our dietary indicators, most of the correlations are statistically non-significant and of relatively small magnitude. Individual components of VAC production system –aquaculture, livestock, and fruit and vegetables– are however found to bear significant correlations with dietary outcomes and could thus indicate that the policy promoting a full VAC system -households producing all of the individual components- might have potentially been over-emphasized.

Our results point to positive associations between dietary indicators and certain specific components of the VAC system, such as with animal protein consumption -through aquaculture- and fiber consumption -through F&V-livestock joint production-, although we do not observe consistent and strong associations to conclusively support a policy towards promoting the complete VAC production system with the purpose of improving dietary outcomes. We find more conclusive evidence of the importance of good access to market and, consequently, Vietnamese policy makers should prioritize connecting remote communities to ensure their market participation for improved nutrition.

Non-separability and endogeneity between production and consumption decisions cannot be ruled out. Indeed, the association we document between adoption of VAC components and household characteristics such as wealth, household size, agricultural land suggests the presence of potential confounding factors intervening in the relationship between VAC adoption and diet quality. As such, our findings should be interpreted as associations, not causal relationships that would require a different study design and data. We recommend that additional empirical research is conducted to establish the overall benefits of VAC adoption before the system is promoted at wide scale as a strategy for promoting improved household dietary diversity and soil quality.

## Data Availability

Permission to use the VLSS data must be obtained from the General Statistical Office in Hanoi, Vietnam. The request should be submitted to: Mr. Nguyen The Quan. Department of Social and Environment Statistics, General Statistical Office, 2, Hoang Van Thu Street, Hanoi, Vietnam, fax: 84–4-846–3511 or 84–4-846–4345, e-mail: ntquan@gso.gov.vn. The request should include a brief (one page) explanation of the proposed research.

## Footnotes

1 Viet Nam’s prevalence of undernourishment (PoU) stood at 6.4% of total population between 2017 and 2019. In comparison, in the same time period neighboring countries such as Cambodia and Laos reported around 14.5% of their population undernourished. Undernourishment is defined as an habitual food consumption insufficient to provide dietary energy levels that are required to maintain a normal, active, and healthy life of an individual (FAO et al., 2020).

2 The PPP conversion factors equalize the nominal exchange rate in terms of the local cost of a common basket of goods and services across countries, for international comparisons of values expressed in nominal currencies.

3 Includes 26 food items such as fragrant rice, sticky rice, beef, buffalo, poultry, etc.

4 Includes 65 food items such as plain rice, maize, potato, wheat grains, beef, buffalo, poultry, peanuts, cabbage, tomato, orange, banana, mango, milk, etc.

5 Accessible here: https://cds.climate.copernicus.eu/#!/home

6 The other crops category includes primarily industrial and staple crops such as soybean, peanuts, sesame, sugarcane, tobacco, cotton, ramie, sedge, tea, coffee, rubber, pepper, coconut, mulberry, cashew, maize, cassava, and so forth.

7 Livestock items include pork, beef, goat, chicken, piglet, duck.

8 Aquaculture items include fish, shrimp.

9 Fruits and vegetables include citrus, pineapple, banana, mango, grape, plum, papaya, jackfruit, potato, morning glory, kohlrabi, cabbage, tomato, beans.

10 The wealth index was constructed using factor analysis (principal-component factor method) following Filmer and Pritchett (2001), based on durable household assets and dwelling condition proxied by the quality of water, toilet, and dwelling construction materials.

11 This figure might be underestimated due to the limited number of fruit and vegetable items collected in the VHLSS 2016 consumption module. Other sources, such as Van Bui et al. (2016), examining different surveys found an average fruit and vegetable consumption of 0.87 and 2.29 servings per day, respectively. These values correspond to approximately 70 g and 183 g of fruit and vegetables consumption per day.

## Code availability

Data processing for this project was undertaken using Stata. Relevant do files can be provided if requested.
